# Clinical Outcomes and Predictors of Improvement With Virtual Behavioral Health Care for Gambling Disorder: Retrospective Cohort Study

**DOI:** 10.2196/101517

**Published:** 2026-07-24

**Authors:** Kelsey McAlister, Elizabeth Knight, Cynthia Grant, Jennifer Huberty

**Affiliations:** 1 Fit Minded, Inc Phoenix, AZ United States; 2 Birches Health New York, NY United States

**Keywords:** behavioral addiction, digital health, industry science, real-world evidence, telehealth

## Abstract

**Background:**

Gambling disorder is associated with substantial psychiatric and functional burden, yet few individuals receive treatment. Limited real-world evidence exists evaluating outcomes of virtually delivered behavioral health care for gambling disorder, particularly among clinically complex patients.

**Objective:**

The purpose of this study was to evaluate gambling symptom severity outcomes among adults with gambling disorder receiving care from Birches Health. We aimed to (1) characterize the clinical profile of adults seeking treatment for gambling disorder; (2) quantify changes in gambling symptom severity over the initial 12 weeks of treatment and examine whether baseline clinical complexity, such as gambling symptom severity, depression severity, and psychiatric comorbidities, was associated with differences in gambling symptom severity improvement over time; and (3) estimate the timing and likelihood of achieving clinically meaningful improvement in gambling symptom severity.

**Methods:**

This retrospective cohort study included 1305 adults receiving virtual behavioral health treatment for gambling disorder through Birches Health between June 2024 and April 2026. Gambling symptom severity was assessed using the Gambling Symptom Assessment Scale (G-SAS) weekly. Linear mixed-effects models evaluated changes in gambling symptom severity over 12 weeks and associations with baseline clinical characteristics. Clinically meaningful improvement was defined as a reduction of 4 or more points in the G-SAS score.

**Results:**

Participants had a mean age of 41.5 (SD 13.1) years, 65.2% (851/1305) were male, and baseline gambling symptom severity was moderate (mean G-SAS score 20.5, SD 11.83). Over half (730/1305, 56%) of participants presented with at least one psychiatric comorbidity, most commonly anxiety disorder (351/1305, 26.9%) and depressive disorder (276/1305, 21.1%). Gambling symptom severity declined significantly over the first 12 weeks of treatment, with G-SAS scores decreasing by approximately 0.099 points per day (*P*<.001), corresponding to an estimated 8.3-point reduction over 12 weeks. Higher baseline depressive symptom severity was associated with faster improvement in gambling symptoms (*P*=.01), whereas depressive disorder (*P*=.03) and attention-deficit/hyperactivity disorder (*P*=.008) diagnoses were associated with slower improvement trajectories. Among patients with routine follow-up assessments recorded during the initial 12 weeks of treatment (1071/1305, 82.1%), 71.7% (935/1305) achieved clinically meaningful improvement in gambling symptom severity, with a median time to improvement of 14 days.

**Conclusions:**

A clinically complex population of adults receiving care through a national virtual behavioral health care provider demonstrated rapid and clinically meaningful reductions in gambling symptom severity. These findings highlight the potential of specialized virtual care models to expand access to gambling treatment and support symptom improvement in routine care settings. Future research should evaluate longer-term recovery trajectories and identify factors associated with sustained improvement and ongoing engagement in care.

## Introduction

Nearly 20 million adults in the United States report engaging in problematic gambling behaviors, and an estimated 2.5 million meet the criteria for a diagnosis of gambling disorder [1]. The consequences of problematic gambling are wide-ranging and serious, including financial distress, relationship breakdown, occupational impairment, and physical and mental health deterioration [2]. Gambling disorder also carries a significantly increased risk of suicide [3]. Despite this substantial burden, treatment seeking remains critically low, with only 20% of affected people having ever sought professional help [4].

Recent policy shifts in the United States, including the legalization of sports betting in 2018, have transformed the gambling landscape and vastly expanded the availability of online wagering [5]. Total sports wagers grew from US $4.9 billion in 2017 to US $121.1 billion in 2023, when 94% of wagers were placed online [5]. As a result of these shifts, internet searches related to help seeking for gambling behavior have increased by 23% since the 2018 ruling [5], suggesting growing public awareness and treatment need. Despite the growing scale of the problem, significant gaps remain in access to effective, evidence-based treatment for gambling disorder.

Psychological intervention, particularly cognitive behavioral therapy (CBT), is currently considered the most effective treatment for gambling disorder, but the broader evidence base remains limited [6]. Most existing studies are small, highly controlled trials, resulting in limited real-world evidence exploring the effectiveness of various treatment modalities in different populations [6]. There is also limited evidence evaluating newer models of care, including virtually delivered treatment, despite growing demand for remote behavioral health services [6]. Additionally, there is no clear consensus within the field on how to measure treatment success, compounding the difficulty in identifying which patients are likely to benefit from treatment [6].

Clinical complexity further complicates treatment for gambling disorder. Over 80% of patients with gambling disorder have a psychiatric comorbidity, including substance use disorder (34%), mood disorders (31%), and anxiety disorder (30%) [7]. Comorbid conditions have been associated with greater gambling symptom severity, higher treatment dropout rates, and poorer treatment outcomes, yet most treatment studies have either excluded patients with comorbidities or lacked sufficient sample sizes to detect their effect, leaving providers with little guidance on how to tailor care for complex presentations [8]. These gaps are particularly acute in virtual care settings, where diverse patient populations seek specialized gambling care at scale. Access to such care has historically been limited by stigma, shame, geographic location, and a scarcity of trained providers [9]. Although there is early evidence suggesting that virtually delivered interventions may reduce gambling-related symptoms, few studies have examined how baseline clinical characteristics relate to treatment outcomes in virtual care settings [10]. As virtual care for gambling addiction treatment continues to expand, understanding which patients improve, how quickly improvement occurs, and how co-occurring psychiatric conditions influence outcomes is critical for evaluating the effectiveness of gambling care in real-world settings.

Birches Health is a national virtual behavioral health care provider specializing in behavioral and process addiction treatment, including care for individuals with gambling disorders. Through a virtual care model (ie, synchronous, video-based outpatient treatment), Birches Health delivers specialized behavioral health care services to patients across the United States, with treatment focused on reducing gambling-related symptoms and addressing co-occurring mental health conditions. Treatment incorporates evidence-based behavioral health approaches commonly used in addiction care, including CBT, motivational interviewing, and relapse prevention strategies. As access to virtual behavioral health treatment continues to expand, evaluating outcomes within real-world settings such as Birches Health helps address important gaps in the existing gambling treatment literature.

The purpose of this study was to evaluate gambling symptom severity outcomes among adults with gambling disorder receiving care from Birches Health. We aimed to (1) characterize the clinical profile of adults seeking treatment for gambling disorder; (2) quantify changes in gambling symptom severity over the initial 12 weeks of treatment and examine whether baseline clinical complexity, such as gambling symptom severity, depression severity, and psychiatric comorbidities, was associated with differences in gambling symptom severity improvement over time; and (3) estimate the timing and likelihood of achieving clinically meaningful improvement in gambling symptom severity. To our knowledge, this is the first study to examine real-world outcomes for gambling disorder treatment delivered virtually in the United States, including the role of baseline clinical complexity in shaping patient response to care.

## Methods

### Study Design and Participants

This study used a retrospective cohort design to evaluate gambling symptom severity outcomes among adults receiving care through Birches Health, a US-based virtual behavioral health care provider that treats behavioral and process addictions. Analyses were conducted using deidentified data collected as part of routine clinical care. For this retrospective study, preregistration of the analysis plan was not applicable. No participant recruitment was conducted for this study as all data were derived from patients receiving care as part of standard clinical services.

The study sample included patients receiving care from Birches Health between June 6, 2024, and April 20, 2026. Patients were included if they (1) were aged 18 years or older; (2) had a documented gambling-related diagnosis based on *ICD-10* (*International Statistical Classification of Diseases, Tenth Revision*), codes corresponding to pathological gambling or gambling-related behaviors; and (3) had at least one recorded assessment of gambling symptom severity. Analyses included all available observations within the 12-week window following each patient’s first recorded assessment. A period of 12 weeks was selected to reflect a clinically meaningful course of outpatient psychotherapy [11,12]. This study was reported in accordance with the STROBE (Strengthening the Reporting of Observational Studies in Epidemiology) guidelines for observational cohort studies (Multimedia Appendix 1).

### Ethical Considerations

The study protocol was reviewed and approved by the Biomedical Research Alliance of New York Institutional Review Board (study ID 26-113-1708). No participant compensation was provided for this research. All patients receiving care through Birches Health provide informed consent as part of the standard clinical onboarding process prior to initiating treatment. This consent covers participation in care, including the scope of services, confidentiality protections, and how their health information may be used and protected. Patients also agree to Birches Health’s privacy policy, which outlines data use practices in accordance with applicable regulations. This study used existing, deidentified clinical data collected during routine care and did not involve any direct interaction with patients. Because the data were deidentified and the research was retrospective in nature, the study qualified for exemption from additional informed consent under US human subject research regulations. Therefore, no additional consent procedures specific to this research were conducted.

### Birches Health Treatment

Birches Health is a privately held, US-based telehealth company founded in 2023 that delivers behavioral health treatment, including for those with gambling-related concerns and other behavioral addictions, to patients in all 50 US states. Care is provided by Master’s-level licensed mental health or addiction counselors (ie, licensed clinical social workers, licensed mental health counselors, licensed marriage and family therapists or licensed alcohol and drug counselors, licensed addiction counselors, and master addiction counselors) with specialized training in gambling disorder treatment through the International Problem Gambling and Gaming Certification Organization. There is no fixed eligibility criterion for treatment; care is provided on a case-by-case basis with flexibility based on individual need. Patients are matched with a specialty provider, including those experienced in the treatment of problem gambling, who is either in-network with the patient’s insurance or eligible for available state grant funding support. Patients may learn about and access Birches Health through a variety of pathways, including direct online search, referrals from health care providers, partnerships with organizations, or other digital channels. Prior to beginning treatment, patients complete clinical paperwork and questionnaires, which are reviewed by the provider during the first appointment as part of the psychiatric diagnostic evaluation. Treatment incorporates evidence-based approaches commonly used in addiction and behavioral health care, including CBT, motivational interviewing, and relapse prevention strategies.

In the first appointment, providers conduct a comprehensive clinical assessment to establish diagnosis, evaluate symptom severity, and identify co-occurring behavioral health conditions. Assessments are based on clinician judgment and information provided on standard measures such as the Gambling Symptom Assessment Scale (G-SAS) and the Patient Health Questionnaire–9 (PHQ-9). On the basis of this assessment, providers collaborate with patients to develop an individualized treatment plan aligned with the patient’s clinical presentation, treatment goals, and preferences. Treatment goals may include reducing gambling-related harms, achieving abstinence from gambling, or improving overall functioning and well-being depending on the patient’s stated objectives. Standardized assessment measures are incorporated into treatment planning and ongoing progress monitoring.

Birches Health delivers a multicomponent, individualized care model through secure video sessions, and the present paper describes symptom trajectories of patients engaging with this care model as implemented in routine clinical practice. Treatment may include individual psychotherapy, group therapy, peer services, financial wellness counseling, and other supportive services targeting the patient’s treatment goals. These may include addressing gambling-related behaviors and other behavioral addictions. When appropriate, treatment may also address co-occurring conditions such as depression, anxiety, substance use disorder, or trauma-related symptoms. Session frequency is determined by clinical need and patient preference, with many patients engaging in care on a weekly or biweekly basis. Duration of care varies depending on treatment progress, symptom severity, and individual goals.

As part of routine care, Birches Health incorporates ongoing symptom monitoring using standardized assessments. Patients complete measures of gambling symptom severity and, in some cases, other mental health symptoms (eg, depression) at a routine cadence throughout treatment. These assessments are integrated into the clinical workflow and used by providers to track progress, inform treatment decisions, and adjust care as needed over time in accordance with measurement-informed care.

### Study Measures

#### Overview

In the initial clinical paperwork, patients report demographic information (eg, age and sex assigned at birth). Providers document the primary clinical diagnosis and any psychiatric comorbidities during the first appointment. As part of routine clinical practice, patients complete standardized symptom assessments. During the study period, gambling symptom severity was assessed on an approximately weekly basis throughout the course of care. Depression symptoms were measured at baseline for all patients and assessed again only at select follow-up time points for some patients.

#### Gambling Symptom Severity

Gambling symptom severity was assessed using the G-SAS, a 12-item self-report measure that evaluates the severity of gambling-related urges, thoughts, behaviors, and associated consequences over the previous week [13,14]. Items assess multiple dimensions of gambling, including urge intensity, frequency and duration of gambling-related thoughts, time spent gambling, emotional distress, and functional impairment. Each item is rated on a 5-point scale from 0 (no symptoms) to 4 (extreme symptoms), yielding total scores ranging from 0 to 48, with higher scores indicating greater symptom severity. The G-SAS has demonstrated good internal consistency in prior research (Cronbach α of approximately 0.85-0.89 [13,14]). The G-SAS has also demonstrated sensitivity to change in prior research [15].

#### Depression

Depressive symptom severity was assessed using the PHQ-9, a 9-item self-report measure evaluating symptoms over the prior 2 weeks [16]. Each item is rated on a 4-point scale ranging from 0 (“not at all”) to 3 (“nearly every day”), yielding total scores from 0 to 27, with higher scores reflecting greater depressive symptom severity. The PHQ-9 has demonstrated high internal consistency (Cronbach α=0.89) [16].

### Statistical Analyses

Baseline demographic and clinical characteristics, including age, biological sex, baseline gambling symptom severity, depression symptom severity, and psychiatric comorbidity, were summarized using descriptive statistics. Continuous variables were reported using means and SDs, and categorical variables were summarized using frequencies and percentages.

To evaluate changes in gambling symptom severity over time, linear mixed-effects models were used to account for repeated observations nested within individuals. Gambling symptom severity (ie, total score on the G-SAS) was modeled as the primary outcome. Analyses were restricted to the first 12 weeks (84 days) of treatment, reflecting a common time frame for short-term outpatient psychotherapy care [11,12] and retaining 64% (835/1305) of the observations across all 1305 patients who met the inclusion criteria. Time was operationalized as days since the first assessment and included as a fixed effect. Models included a random intercept for each patient to account for within-person correlation across repeated assessments. All models were adjusted for age and biological sex. To examine whether baseline clinical characteristics were associated with differences in gambling symptom severity trajectories, mixed-effects models were extended to include baseline predictors as fixed effects, including baseline gambling symptom severity (ie, G-SAS total score), depression symptom severity (ie, PHQ-9 total score), and psychiatric comorbidities. Baseline gambling symptom severity was included as a covariate to account for potential bias introduced by the use of an absolute change threshold given that patients with higher baseline scores have greater room for improvement. Psychiatric comorbidities were grouped by clinical category, and the 5 most prevalent groups (anxiety disorder, depressive disorder, trauma- and stress-related disorders, substance use disorder, and attention-deficit/hyperactivity disorder [ADHD]), which are also commonly reported among individuals with gambling disorder in prior literature [7,17], were included in the models. Continuous predictors (time, age, baseline G-SAS score, and baseline PHQ-9 score) were standardized prior to fitting the extended model to facilitate comparison of effect sizes across predictors with different scales. Interaction terms between baseline predictors and time were included to evaluate whether rates of change differed across subgroups.

To assess clinically meaningful improvement in gambling symptom severity, change in G-SAS total score from baseline was calculated at each follow-up assessment during the 12-week study period. Clinically meaningful improvements were defined as a reduction of at least 4 points on the G-SAS, consistent with prior literature [15]. Time-to-improvement analyses were conducted by identifying the earliest time point at which each patient met this threshold. Patients who did not meet the criteria for improvement during the following 12 weeks were treated as censored observations.

All models were estimated using restricted maximum likelihood, allowing for inclusion of all available observations under the assumption of being missing at random. No imputation procedures were applied. Model assumptions were evaluated graphically via residual plots and *Q*-*Q* plots. Residuals showed evidence of heteroscedasticity and heavy tails, consistent with the bounded nature of the G-SAS scale; these violations are common in real-world clinical data and were unlikely to substantially affect the conclusions. A 2-sided *P* value below .05 was used throughout. All analyses were conducted in R (version 4.4.2; R Foundation for Statistical Computing) using the *lme4* and *lmerTest* packages.

### Bias Mitigation

To mitigate potential conflicts of interest, the following safeguards were applied: (1) research questions were developed by the scientific team based on scientific merit and were not prescribed by Birches Health, (2) study outcomes were not guaranteed as contractual deliverables, (3) the analysis plan was prespecified prior to data access, and (4) data analysis and interpretation were led by the authors operating independently of Birches Health. No author’s employment status or compensation is contingent on the direction or outcome of the study.

## Results

### Descriptive Characteristics

A total of 12,336 Birches Health patient records were screened for inclusion, of which 1305 (10.6%) met the inclusion criteria (excluding n=10,594, 85.9% without an eligible gambling-specific diagnosis; n=3, 0% under the age of 18 years; and n=434, 3.5% without a documented G-SAS assessment). The mean number of sessions attended was 12.88 (SD 11.56; median 9; range 1-76). The mean time in care was 95.27 (SD 81.12; median 70) days. The mean age was 41.5 (SD 13.1) years. The sample was predominantly male (851/1305, 65.2%). Baseline gambling symptom severity was moderate, with a mean G-SAS score of 20.5 (SD 11.83). Depressive symptom severity was also moderate at baseline, with a mean PHQ-9 score of 11.12 (SD 6.46; Table 1). Psychiatric comorbidities were frequently observed alongside gambling disorder, with over half (730/1305, 56%) of the sample presenting with at least one additional diagnosis. The most prevalent comorbidities were anxiety disorder (351/1305, 26.9%), depressive disorder (276/1305, 21.1%), trauma- and stress-related disorders (125/1305, 9.6%), substance use disorder (85/1305, 6.5%), and ADHD (66/1305, 5.1%). Of the 1305 patients, 1071 (82.1%) had at least one follow-up G-SAS assessment in the following 12 weeks. Patients with and without follow-up data did not differ significantly on any baseline demographic or clinical characteristics examined (Multimedia Appendix 2).

**Table 1:** Patients’ descriptive and clinical characteristics (N=1305).

			Values
Age (y), mean (SD)			41.46 (13.05)
Baseline PHQ-9^a^ score (0-27), mean (SD)			11.12 (6.46)
Baseline G-SAS^b^ score (0-48), mean (SD)			20.5 (11.83)
**Gender, n (%)**			
	Female	398 (30.5)	
	Male	851 (65.2)	
	Transgender or nonbinary	3 (0.2)	
	Unknown	52 (4)	
**Psychiatric comorbidities, n (%)**			
	Anxiety disorder	351 (26.9)	
	Depressive disorder	276 (21.1)	
	Trauma- and stress-related disorders	125 (9.6)	
	Substance use disorder	85 (6.5)	
	ADHD^c^	66 (5.1)	
	Other	59 (4.5)	
	Bipolar disorder	33 (2.5)	
	Impulse control disorders	22 (1.7)	
	OCD^d^-related disorders	17 (1.3)	

^a^PHQ-9: Patient Health Questionnaire–9.

^b^G-SAS: Gambling Symptom Assessment Scale.

^c^ADHD: attention-deficit/hyperactivity disorder.

^d^OCD: obsessive-compulsive disorder.

### Gambling Symptom Severity Trajectories Over Time

Gambling symptom severity declined significantly over the course of treatment. In an initial model adjusting for age and biological sex, G-SAS scores decreased by approximately 0.099 points per day (*P*<.001), corresponding to a reduction of roughly 2.8 points over 4 weeks and 8.3 points over 12 weeks. Age was not significantly associated with gambling symptom severity trajectories (*P*=.16). Female individuals presented with significantly higher baseline gambling symptom severity than male individuals (β=4.44; *P*<.001). Model fit statistics indicated a low marginal *R*^2^ (0.082) and moderate conditional *R*^2^ (0.663), reflecting that time, age, and biological sex alone explained little of the variance in gambling symptom severity, with most of the explained variance attributable to substantial between-person differences.

To examine whether baseline clinical characteristics were associated with differences in gambling symptom severity trajectories, the model was extended to include baseline G-SAS score, baseline PHQ-9 score, and 5 categories of psychiatric comorbidities (anxiety disorder, depressive disorder, trauma- and stress-related disorders, substance use disorder, and ADHD) as fixed effects, along with their interactions with time. Time remained a significant negative predictor of gambling symptom severity (ie, symptoms decreased over time) after accounting for these characteristics (*P*<.001), and age remained nonsignificant (*P*=.52). The effect of female sex on baseline severity was attenuated relative to the initial model (β=1.23 vs 4.44), suggesting that baseline clinical characteristics accounted for a portion of this difference.

Baseline depression symptom severity showed a marginal negative association with overall gambling symptom severity (*P*=.05) and a significant negative interaction with time (*P*=.01), suggesting that patients with higher baseline depression symptom burden showed faster improvement in gambling symptoms over the 12-week window. Among psychiatric comorbidity categories, a depressive disorder diagnosis was associated with significantly higher baseline gambling symptom severity (*P*=.02) and slower improvement over time (*P*=.03). ADHD showed the largest interaction effect, with patients showing slower improvement over time (*P*=.008). Notably, patients with ADHD presented with significantly lower baseline gambling symptom severity than those without (β=−3.019; *P*=.02), suggesting a distinct clinical profile. Substance use disorder was associated with significantly higher baseline gambling symptom severity (*P*=.002) but did not show a significant interaction with time (*P*=.10), indicating that, while these patients presented with greater severity, their rate of improvement did not differ significantly from those without a substance use diagnosis. Trauma- and stress-related disorders and anxiety disorder were not significantly associated with baseline gambling symptom severity or rate of change within the 12-week window.

The addition of baseline clinical characteristics, including diagnosis and symptom severity, substantially improved the fixed-effects explanation of gambling symptom severity trajectories (marginal *R*^2^=0.082 to 0.538), whereas the conditional *R*^2^ remained stable (0.663 to 0.690), suggesting that clinical variables accounted for variance previously attributable to between-person differences. The full model results are shown in Table 2. Trajectory model results were consistent when analyses were restricted to patients with at least one follow-up assessment (marginal *R*^2^=0.082; conditional *R*^2^=0.661), supporting the robustness of the findings to potential attrition bias (Multimedia Appendix 2).

**Table 2:** Linear mixed-effects models of gambling symptom severity over time^a^.

				β (SE; 95% CI)		*P* value
**Model 1: time, age, and biological sex^b^**						
	**Fixed effects**					
		Time (days since first assessment)	−.099 (.004; –.107 to –.092)		<.001	
		Age	.034 (.024; –.014 to .081)		.16	
	**Biological sex (reference=male)**					
		Female	4.437 (.688; 3.089 to 5.785)		<.001	
		Unknown	1.917 (1.466; –.957 to 4.791)		.19	
**Model 2: baseline clinical characteristics added^c^**						
	**Fixed effects**					
		Time (standardized)	−11.522 (.545; –12.591 to –10.453)		<.001	
		Age (standardized)	.120 (.186; –.245 to .485)		.52	
	**Biological sex (reference=male)**					
		Female	1.229 (.414; .417 to 2.040)		.003	
		Unknown	.126 (.900; –1.638 to 1.890)		.89	
	**Baseline clinical characteristics**					
		G-SAS^d^ total score (standardized)	4.146 (.303; 3.552 to 4.740)		<.001	
		PHQ-9^e^ total score (standardized)	−.606 (.308; –1.210 to –.002)		.05	
	**Psychiatric comorbidities (reference=absent)**					
		Anxiety disorder	−.931 (.716; –2.334 to .472)		.19	
		Depressive disorder	1.792 (.777; .270 to 3.314)		.02	
		Trauma- and stress-related disorders	.918 (.983; –1.008 to 2.844)		.35	
		Substance use disorder	3.268 (1.059; 1.192 to 5.344)		.002	
		ADHD^f^	−3.019 (1.271; –5.510 to –.528)		.02	
	**Interactions with time**					
		Time × G-SAS baseline	−6.963 (.414; –7.775 to –6.151)		<.001	
		Time × PHQ-9 baseline	−1.066 (.424; –1.897 to –.235)		.01	
		Time × anxiety disorder	−1.199 (.988; –3.135 to .738)		.23	
		Time × depressive disorder	2.280 (1.072; .179 to 4.381)		.03	
		Time × trauma- and stress-related disorders	1.080 (1.363; –1.591 to 3.751)		.43	
		Time × substance use disorder	2.400 (1.463; –.467 to 5.267)		.10	
		Time × ADHD	−4.701 (1.763; –8.156 to –1.246)		.008	

^a^Outcome is Gambling Symptom Assessment Scale total score. Analyses were restricted to the first 12 weeks (84 days) of treatment. Model 1: N=1305 patients; 6547 observations. Model 2: n=1293 patients; 6518 observations; the reduction in sample size relative to model 1 reflects missing baseline Patient Health Questionnaire–9 data (n=12). In model 2, continuous predictors (time, age, baseline Gambling Symptom Assessment Scale score, and baseline Patient Health Questionnaire–9 score) were standardized prior to model fitting. Gender/biological sex minority groups were omitted due to small cell sizes.

^b^Random effects: between-person variance=84.67% and residual variance=49.13%; model fit: marginal *R*^2^=0.082 and conditional *R*^2^=0.663.

^c^Random effects: between-person variance=22.20% and residual variance=45.28%; model fit: marginal *R*^2^=0.538 and conditional *R*^2^=0.690.

^d^G-SAS: Gambling Symptom Assessment Scale.

^e^PHQ-9: Patient Health Questionnaire–9.

^f^ADHD: attention-deficit/hyperactivity disorder.

### Time to Clinically Meaningful Improvement

Among patients with at least one follow-up G-SAS assessment within 12 weeks (1071/1305, 82.1%), 71.7% (935/1305) achieved a clinically meaningful reduction in gambling symptom severity during treatment (≥4 points), with a median time to improvement of 14 (mean 19.3, SD 16.4) days. In a sensitivity analysis requiring improvement to be sustained at a consecutive assessment, the sustained improvement rate was 47.7% (622/1305), with a median time to sustained improvement of 14 (mean 19.4, SD 15.6) days.

To contextualize these findings within the broader gambling symptom severity trajectories observed across the full cohort, the time-to-improvement analysis provides a complementary individual-level perspective on symptom changes. While the trajectory analysis estimated an average reduction of 2.8 points over 4 weeks at the population level, among patients who achieved clinically meaningful improvement, the median time to reaching this threshold was just 14 days. The slower population-level trajectory estimate reflects averaging across all patients regardless of treatment response, which attenuates the observed magnitude of change relative to those who improved.

## Discussion

### Principal Findings

The purpose of this study was to evaluate gambling symptom severity outcomes among adults with gambling disorder receiving care from Birches Health. We aimed to (1) characterize the clinical profile of adults seeking treatment for gambling disorder; (2) quantify changes in gambling symptom severity over time and examine whether baseline clinical complexity, such as gambling symptom severity, depression severity, and psychiatric comorbidities, was associated with differences in gambling symptom severity improvement over time; and (3) estimate the timing and likelihood of achieving clinically meaningful improvement in gambling symptom severity. Adults entering treatment presented with a mean baseline gambling symptom severity in the moderate range; moderate depressive symptoms; and high rates of co-occurring psychiatric diagnoses, most commonly anxiety, depressive, and trauma- and stress-related disorders. Gambling symptom severity declined significantly over the first 12 weeks of treatment, and baseline clinical characteristics, including gambling symptom severity, depressive symptoms, and comorbid diagnoses, differentially shaped trajectories of change. Nearly three-quarters of patients (935/1305, 71.7%) achieved a clinically meaningful reduction in gambling symptom severity during their course of treatment, with a median time to improvement of 14 days.

This clinical profile is consistent with prior literature on adults seeking treatment for gambling disorder. High rates of co-occurring mood disorders (30.9%) and anxiety disorder (29.9%) are well documented in this population, and treatment-seeking samples are consistently male predominant, although prevalence among women is rising [7,18]. Women with gambling disorder face well-documented barriers to treatment, including shame, stigma, gender role expectations, and a lack of social support, and may perceive these barriers more acutely than men [19]. The meaningful representation of women in this sample (398/1305, 30.5%) is therefore notable and may reflect the accessibility of a virtually delivered care model that reduces some of these structural and social barriers to help seeking. The availability of gender-or biological sex–specific programs within this model, including a women’s gambling support group, may have further supported engagement among women [20].

More broadly, the present findings suggest that virtually delivered behavioral health treatment may successfully engage and retain a clinically complex population, including those with psychiatric comorbidities. There is current evidence also suggesting that psychiatric comorbidities are not a contraindication to most treatments for gambling disorder [21]. This study’s sample included rates of mood, anxiety, and substance use disorders consistent with those reported in the literature, and there is current evidence suggesting bidirectional relationships between gambling and comorbid conditions [18]. The engagement of individuals with moderate symptom severity and high rates of psychiatric comorbidities suggests that virtually delivered care is not limited to individuals with less complex clinical presentations and may represent an accessible treatment option for a broad range of patients seeking gambling-related care.

Gambling symptom severity declined significantly over 12 weeks, with an estimated average reduction of 8.3 points on the G-SAS. This is consistent with evidence that digitally delivered, evidence-based treatment produces symptom reductions comparable to those of face-to-face care [10]. One study reported an even larger effect on the G-SAS for a structured digital CBT intervention in a smaller sample; our study’s large sample and naturalistic setting extend the evidence to real-world treatment contexts [10]. Several characteristics of virtually delivered behavioral health care may plausibly contribute to gambling symptom severity improvement, including reduced geographic and logistical barriers to care and lower perceived stigma associated with help seeking, both of which have been identified as major barriers to gambling treatment engagement [21,22]. Access to providers experienced in treating behavioral addictions may also be important given evidence that specialized gambling treatment is often limited and difficult to access within traditional behavioral health settings [23]. Together, these findings further support the growing evidence base for virtually delivered behavioral health treatment (such as that offered by Birches Health) for gambling disorder. Future studies should examine which specific components of virtual behavioral health care, including factors related to accessibility, stigma reduction, and provider specialization, are most strongly associated with treatment engagement and sustained symptom improvement. The gap observed between initial and sustained improvement rates in this study (935/1305, 71.7% vs 622/1305, 47.7%, respectively) suggests that maintaining early gains may be a particular challenge in this population and that virtual care models may benefit from incorporating more structured or phase-based approaches to support longer-term recovery.

Notably, higher depression symptom severity at the start of care, as measured via PHQ-9 scores, was associated with faster improvement in gambling symptom severity. One possible explanation is that elevated depressive symptoms may partly reflect acute distress related to gambling-related consequences such as financial strain, interpersonal conflict, or loss of control, which may improve relatively quickly as gambling behavior stabilizes [12]. The integration of individualized treatment planning and concurrent treatment of gambling-related and co-occurring mental health concerns within the Birches Health care model may have further supported improvement among patients presenting with elevated depressive symptoms at baseline, although causal conclusions cannot be drawn from this observational study. However, in contrast to elevated depressive symptoms alone, the presence of a depressive disorder diagnosis predicted slower improvement trajectories, possibly reflecting a more chronic or recurrent condition that may require more intensive or longer-term intervention [24].

Similarly, comorbid ADHD and substance use disorder each reflected clinically distinct presentations that meaningfully influenced gambling symptom severity trajectories. Patients with substance use disorder presented with significantly higher baseline gambling symptom severity, whereas patients with ADHD demonstrated slower improvement over time. These findings further underscore the heterogeneity of individuals seeking treatment for gambling disorder and reinforce the importance of treatment models that address gambling-related behaviors within the broader context of co-occurring psychiatric and behavioral health needs rather than treating gambling in isolation [24,25]. Birches Health’s virtually delivered behavioral health model may address this complexity, offering patients access to providers with specialized training in behavioral and process addictions who can assess and treat co-occurring conditions alongside gambling disorder within a single, accessible care setting. Patients with greater psychiatric complexity may also benefit from more structured and coordinated models of care, such as those incorporating phase-based progression and ongoing behavioral monitoring, to support sustained recovery outcomes [26,27]. Virtual behavioral health models such as Birches Health may represent a scalable approach to delivering this type of care within gambling treatment settings, and future research should evaluate whether incorporating more structured and phase-based models of care delivered by providers with specialized training in the treatment of gambling disorder improves outcomes among patients with co-occurring psychiatric conditions.

The median time to clinically meaningful improvement in gambling symptom severity was 14 days, indicating that improvement was both rapid and substantial for patients who engaged with care. Nearly three-quarters of patients with follow-up data (935/1305, 71.7%) achieved clinically meaningful improvement. Prior studies of CBT and internet-delivered interventions for gambling disorder have demonstrated significant reductions in gambling-related symptoms in both routine addiction care and randomized trials [10,28], although few have examined the timing of improvement. Notably, one randomized trial observed that the largest reduction in gambling symptom severity occurred early in treatment, between the initial assessment and treatment baseline, which is consistent with the rapid early improvement observed in this study [28]. This improvement may reflect the high frequency of patient contact and ongoing symptom monitoring embedded within this model of care, but achieving sustained improvement remains a challenge for many patients. Future research should examine how early symptom improvement relates to longer-term recovery trajectories, including sustained engagement, relapse risk, and progression through different phases of recovery over time.

### Strengths and Limitations

This study has several strengths. To our knowledge, this is the first large-scale, naturalistic study to examine outcomes of synchronous, video-based behavioral health care for gambling disorder. The large, real-world sample of 1305 adults with gambling disorder receiving care through a nationally operating virtual provider enhances generalizability and supports high external validity. The use of a validated outcome measure (G-SAS) facilitates benchmarking against other treatment contexts, and the longitudinal mixed-effects modeling approach used all available observations, providing a more complete picture of symptom change than complete-case designs. This study contributes to a limited but growing literature evaluating virtually delivered behavioral health treatment for gambling disorder, particularly within US-based routine care settings.

Several limitations should be noted. First, the observational design without a control condition precludes causal inference as observed improvements may reflect treatment effects, natural recovery, regression to the mean, or some combination of these. Future studies incorporating comparison conditions would strengthen causal conclusions. Second, follow-up assessments varied across patients and were not completed consistently over time, particularly for measures of co-occurring mental health symptoms such as depression symptoms. As a result, this limited examination of mental health symptom trajectories. This also introduced potential bias in PHQ-9–related model estimates. Future studies should incorporate more consistent longitudinal assessment of co-occurring mental health symptoms to better understand how changes in psychiatric functioning relate to gambling recovery over time. However, patients in this study with and without follow-up gambling symptom severity assessment did not differ significantly on baseline demographic or clinical characteristics. Third, analyses were limited to the first 12 weeks of treatment and, therefore, do not capture long-term recovery trajectories for patients who remained in care. Fourth, clinically meaningful improvement analyses were limited to patients with at least one follow-up assessment as 17.9% (234/1305) of the sample did not complete follow-up gambling symptom severity measures. Although patients with and without follow-up assessments did not differ on baseline characteristics, attrition may have biased estimates of treatment response. Future studies should examine patterns of disengagement and incorporate strategies to improve longitudinal assessment completion in routine care settings. Fifth, this study also did not account for potential therapist-level effects, concurrent pharmacological treatment for gambling disorder, or individual treatment intervention components, which may have influenced gambling symptom severity trajectories. Future research should examine how provider characteristics and pharmacological treatment relate to outcomes in virtual gambling disorder care and which components of the care model are most effective. Sixth, the finding regarding higher depressive symptom severity being associated with faster gambling symptom improvement whereas a depressive disorder diagnosis predicted slower improvement trajectories should be interpreted cautiously given the observational nature of the study and potential heterogeneity in depressive presentations. Future studies should examine how chronicity, severity, and subtypes of depressive symptoms differentially relate to gambling recovery over time. Finally, gender/biological sex minority groups were underrepresented, and race and ethnicity data were unavailable, limiting conclusions about equity in outcomes.

### Conclusions

This study demonstrated that adults with gambling disorder receiving virtually delivered behavioral health care achieved rapid, clinically meaningful reductions in gambling symptom severity, with nearly three-quarters of patients (935/1305, 71.7%) reaching a clinically meaningful threshold within a median of 14 days. Baseline clinical characteristics, including gambling symptom severity, depressive symptoms, and psychiatric comorbidities, differentially shaped trajectories of improvement; yet, meaningful gains were observed across a range of clinical profiles, underscoring the capacity of this care model to effectively serve a complex and heterogeneous population. These findings address a meaningful gap in the literature by providing large-scale, naturalistic evidence that virtually delivered care for gambling disorder is associated with clinically meaningful symptom improvement in a clinically complex population. Future research should evaluate long-term recovery trajectories and the role of specialized virtual behavioral health care in supporting patients with gambling disorder across diverse clinical profiles.

## Data Availability

The dataset used for this retrospective cohort analysis is not publicly available given Birches Health’s privacy policy for patient data. However, aggregated and anonymized data may be shared with interested parties on reasonable request.

## Appendix

Multimedia Appendix 1 STROBE checklist.

Multimedia Appendix 2 Statistical analysis.

## Abbreviations

ADHD: attention-deficit/hyperactivity disorder
CBT: cognitive behavioral therapy
G-SAS: Gambling Symptom Assessment Scale
ICD-10: International Statistical Classification of Diseases, Tenth Revision
PHQ-9: Patient Health Questionnaire–9
STROBE: Strengthening the Reporting of Observational Studies in Epidemiology
