# Supplementary material for: Clinical Outcomes and Predictors of Improvement With Virtual Behavioral Health Care for Gambling Disorder: Retrospective Cohort Study
Source: JMIR Ment Health. 2026 Jul 24;13:e101517. doi: 10.2196/101517 (PMC13451867; doi:10.2196/101517)
Supplement: Supplementary file 2 [file mental_v13i1e101517_app2.docx]

**Supplemental Material 2. Sensitivity Analysis**

**Baseline Characteristics by Follow-up Assessment Availability.**

|  | **Baseline Only**  **(n = 234)** | **Has Follow-up**  **(n = 1,071)** | **p** | **SMD** |
| --- | --- | --- | --- | --- |
| **Age, mean (SD)** | 40.15 (13.07) | 41.75 (13.04) | .091 | 0.122 |
| Gender, n (%) | | | | |
| **Female** | 78 (33.3) | 320 (29.9) | .730 |  |
| **Male** | 146 (62.4) | 705 (65.8) |  |  |
| **Transgender or nonbinary** | 0 (0.0) | 3 (0.3) |  |  |
| **Unknown** | 0 (0.0) | 1 (0.1) |  |  |
| **Baseline G-SAS, mean (SD)** | 20.44 (11.86) | 20.52 (11.83) | .926 | 0.007 |
| **Baseline PHQ-9, mean (SD)** | 11.38 (6.63) | 11.07 (6.42) | .520 | 0.048 |

*Note. Baseline Only = patients with a single G-SAS assessment; Has Follow-up = patients with at least one follow-up G-SAS assessment. Group differences were assessed using independent samples t-tests for continuous variables and chi-square tests for categorical variables. SMD = standardized mean difference.*

**Gambling Symptom Severity Trajectory Model Fit: Full Sample vs. Patients with Follow-up Assessments.**

|  | **Full Sample**  **(n = 1,305)** | **≥2 Assessments**  **(n = 1,071)** |
| --- | --- | --- |
| **Marginal R²** | 0.082 | 0.082 |
| **Conditional R²** | 0.663 | 0.661 |
| **ICC** | 0.633 | 0.631 |
| **RMSE** | 6.397 | 6.460 |

*Note. Full Sample = all patients meeting inclusion criteria (N = 1,305); ≥2 Assessments = patients with at least one follow-up G-SAS assessment (n = 1,071). Both models include time, age, and gender as fixed effects with a random intercept for each patient. ICC = intraclass correlation coefficient; RMSE = root mean square error.*
